# Current status of postoperative infections after digestive surgery in Japan: The Japan Postoperative Infectious Complications Survey in 2015

**DOI:** 10.1002/ags3.12236

**Published:** 2019-03-12

**Authors:** Toru Niitsuma, Shinya Kusachi, Yoshio Takesue, Hiroshige Mikamo, Koji Asai, Manabu Watanabe

**Affiliations:** ^1^ Department of Surgery Toho University Graduate School of Medicine Tokyo Japan; ^2^ Department of Surgery Toho University Ohashi Medical Center Tokyo Japan; ^3^ Department of Infection Prevention and Control Hyogo College of Medicine Hyogo Japan; ^4^ Department of Clinical Infectious Diseases Aichi Medical University Aichi Japan

**Keywords:** nosocomial infection, postoperative infection, remote infection, surgical site infection, surveillance

## Abstract

**Aim:**

To survey postoperative infections (PI) after digestive surgery.

**Methods:**

This survey, conducted by the Japan Society of Surgical Infection, included patients undergoing digestive surgery at 28 centers between September 2015 and March 2016. Data collected included patient background characteristics, type of surgery, contamination status, and type of PI, including surgical site infection (SSI), remote infection (RI), and antimicrobial‐resistant (AMR) bacterial infections and colonization.

**Results:**

Postoperative infections occurred in 10.7% of 6582 patients who underwent digestive surgery (6.8% for endoscopic surgery and 18.7% for open surgery). SSI and RI, including respiratory tract infection, urinary tract infection, antibiotic‐associated diarrhea, drain infection, and catheter‐related bloodstream infection, occurred in 8.9% and 3.7% of patients, respectively. Among all PI, 13.2% were overlapping infections. The most common overlapping infections were incisional and organ/space SSI, which occurred in 4.2% of patients. AMR bacterial infections occurred in 1.2% of patients after digestive surgery and comprised 11.5% of all PI. Rate of AMR bacterial colonization after digestive surgery was only 0.3%.

**Conclusion:**

Periodic surveillance of PI, including AMR bacteria, is necessary for a detailed evaluation of nosocomial infections.

## 1 INTRODUCTION

Data collection, analysis, and feedback are necessary to reduce the incidence of postoperative infections (PI), which are a significant source of postoperative complications and an important problem for all healthcare workers.^1^ Surgical site infections (SSI) are common, accounting for 38% of all postoperative complications,^2^ with an incidence rate ranging between 4.0% and 24%.^3^, ^4^, ^5^, ^6^, ^7^, ^8^, ^9^, ^10^ Additionally, SSI are associated with longer hospital stays, higher rates of reoperation and readmission, as well as increased medical costs and mortality rates.^10^, ^11^ Therefore, comprehensive understanding of the current status of SSI is necessary for effective PI control in Japan.

Remote infections (RI) can occur at various sites after surgery,^5^ such as the respiratory and urinary tracts, as well as drain infection, and include antibiotic‐associated diarrhea and catheter‐related bloodstream infection.^12^ In certain cases, RI can be associated with antimicrobial‐resistant (AMR) bacterial infection and colonization, which have become an increasingly significant problem, with increased global incidence and emergence among community‐acquired infections.^9^, ^13^, ^14^, ^15^, ^16^ Therefore, investigation of the prevalence of RI as well as AMR bacterial infections and colonization is important for better understanding of this global problem.

Herein, we present the results of the Japan Postoperative Infectious Complications Survey in 2015 (JPICS‐15), which examined the incidences and types of PI after digestive surgery. We also determined the rates of specific PI, including SSI and RI as well as AMR bacterial infections and colonization.

## 2 METHODS

This voluntary survey was conducted by the Japan Society for Surgical Infection at 28 centers, which included 16 university hospitals, 11 general hospitals, and one cancer center. A detailed list of the participating facilities is given in the Acknowledgments section. Patient data (ie, age, gender, type of surgical procedure, contamination status, PI, isolated bacteria, presence and status of AMR bacteria, and prognosis) were prospectively accumulated between September 2015 and March 2016. The observational period was 30 days after surgery or until discharge. Data regarding PI were recorded in an online surveillance database (https://entry3.eps.co.jp/infection_svlce/Password/PasswordReSet.aspx). Direct input was needed only for the date of surgery and patient age, whereas other parameters were entered using pull‐down menus. For patients without PI, only age, gender, and surgical procedure were submitted.

Surgical procedures included esophageal surgery, gastrointestinal surgery, colorectal surgery, liver surgery, biliary surgery, cholecystectomy, pancreatic surgery, appendectomy, hernia surgery, and surgery for acute peritonitis. In addition, the surgical procedures were categorized as open or endoscopic. Contamination status of patients with PI was categorized as class I (clean), II (clean‐contaminated), III (contaminated), or IV (dirty‐infected), depending on the surgical conditions, according to the Centers for Disease Control and Prevention wound classification guidelines.^17^

Postoperative infections were defined as incisional and organ/space SSI and RI, which included respiratory tract infections (RTI), urinary tract infections (UTI), antibiotic‐associated diarrhea, catheter‐related bloodstream infections, drain infections, and bacteremia of unknown origin. All SSI, including incisional and organ/space SSI, were defined according to the National Healthcare Safety Network of the Centers for Disease Control and Prevention.^17^ Confirmation of bacterial infection was based on the isolation of bacteria from specimens with inflammatory findings of high fever and elevated white blood cell counts and C‐reactive protein levels, whereas bacterial colonization was defined as the presence of low‐volume bacteria, such as more than 1+ by qualitative analysis, in the absence of any inflammatory findings.

Data were collected to determine the infection and colonization status for the following AMR bacteria: *Clostridioides difficile* colitis (CD colitis), methicillin‐resistant *Staphylococcus aureus* (MRSA), extended‐spectrum beta‐lactamase (ESBL)‐producing Enterobacteriaceae, imipenem‐resistant *Pseudomonas aeruginosa* (IPM‐RP), vancomycin‐resistant enterococci (VRE), and multidrug‐resistant Gram‐negative bacteria (MDR‐GN).

## 3 RESULTS

The study cohort included 6582 patients who underwent digestive surgery during the study period, which included 279 esophageal surgeries (open: 243; endoscopic: 36), 975 gastrointestinal surgeries (open: 467; endoscopic: 508), 1724 colorectal surgeries (open: 548; endoscopic: 1176), 497 liver surgeries (open: 490; endoscopic: 7), 76 biliary surgeries (open: 68; endoscopic: 8), 1224 cholecystectomies (open: 120; endoscopic: 1104), 295 pancreatic surgeries (open: 295; endoscopic: 0), 545 appendectomies (open: 123; endoscopic: 422), 821 hernia surgeries, and 146 surgeries for acute peritonitis (Table 1). Hernia surgery and surgery for acute peritonitis were not categorized as open or endoscopic. In total, 2354 patients (35.8%) who underwent open surgery and 3261 (49.5%) who underwent endoscopic surgery were included in the survey.

**Table 1:** Distribution of surgical procedures

Surgical procedure	Total	Open	Endoscopic
Esophageal surgery	279	243	36
Surgery for esophageal cancer	191	191	0
Surgery for esophageal hiatal hernia	19	0	19
Surgery for esophageal achalasia	17	1	16
Esophagectomy and reconstruction	16	16	0
Esophageal dilation	14	14	0
Two‐stage operation for reconstruction after esophagectomy	9	9	0
Esophageal tumor resection	7	7	0
Surgery for esophageal varices	2	2	0
Drainage of periesophageal abscess	2	2	0
Esophageal diverticulectomy	1	1	0
Selective vagotomy	1	0	1
Gastrointestinal surgery	975	467	508
Gastrectomy	525	161	364
Total gastrectomy	236	167	69
Partial gastrectomy	62	13	49
Gastroenterostomy	58	58	0
Gastrorrhaphy	30	30	0
Proximal gastrectomy	24	24	0
Gastrorrhaphy/duodenorrhaphy for perforated ulcer	22	0	22
Duodenojejunostomy	7	7	0
Pyloroplasty	5	2	3
Gastroduodenal diverticulectomy/polypectomy	3	3	0
Fundoplication	2	1	1
Gastric vascular ligation	1	1	0
Colorectal surgery	1724	548	1176
Colectomy for colon cancer	487	0	487
Partial colectomy/hemicolectomy	467	198	269
Low anterior resection	351	96	255
Total/subtotal colectomy	177	156	21
Rectal amputation	118	50	68
Rectal resection	103	27	76
Colon tumor resection/diverticulectomy/polypectomy	12	12	0
Super‐low anterior resection	9	9	0
Liver surgery	497	490	7
Hepatectomy	489	489	0
Fenestration for hepatic cyst	7	0	7
Hepatorrhaphy	1	1	0
Biliary surgery	76	68	8
Choledochogastrostomy (Choledochojejunostomy)	22	22	0
Surgery for bile duct cancer	20	20	0
Choledocholithotomy	16	8	8
Surgery for gallbladder cancer	13	13	0
Surgery for biliary dilatation	2	2	0
Incision or plication for biloma	2	2	0
Intrahepatic cholangiogastrostomy (Cholangiojejunostomy)	1	1	0
Cholecystectomy	1224	120	1104
Pancreatic surgery	295	295	0
Pancreatoduodenectomy	137	137	0
Distal pancreatectomy	127	127	0
Total pancreatectomy	13	13	0
Pancreaticojejunostomy	10	10	0
Pancreatoduodenectomy for gallbladder cancer	8	8	0
Appendectomy	545	123	422
Hernia surgery	821		
Surgery for acute peritonitis	146		
Total	6582	2354	3261

**Figure 1:**
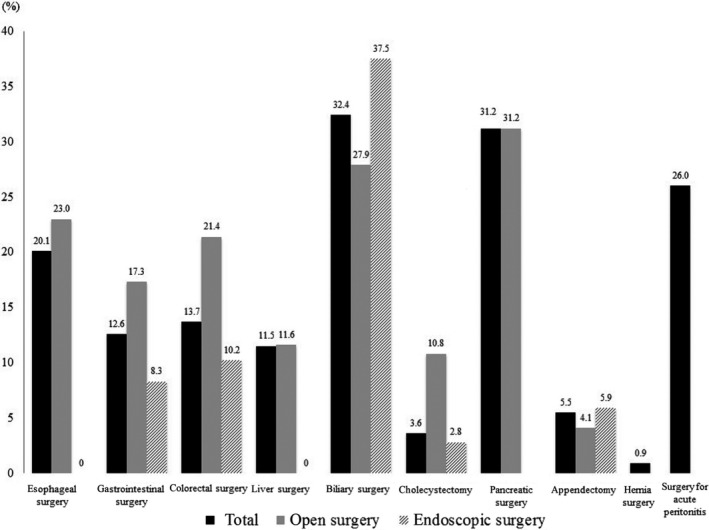
Incidence of postoperative infections according to the surgical procedure types

In total, 706 (10.7%) patients (481 males and 225 females; median age, 63 years; age range, 10–90 years) developed PI. In regard to contamination status, 25 cases were class I, 582 class II, 43 class III, and 56 class IV. PI occurred in 440 (18.7%) patients who underwent open surgery and in 221 (6.8%) who underwent endoscopic surgery. Figure 1 shows the incidence of PI according to the type of surgery and included the total number, open and endoscopic surgeries.

**Figure 2:**
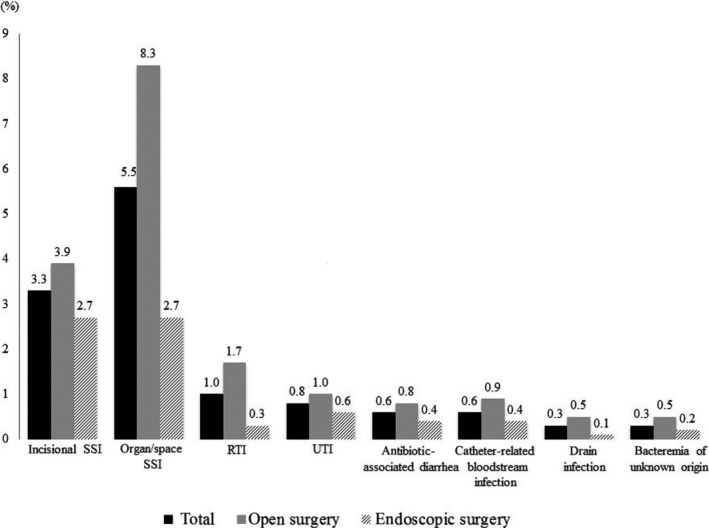
Details of postoperative infections according to the type of surgery. RTI, respiratory tract infection; SSI, surgical site infection; UTI, urinary tract infection

Of all PI, SSI and RI accounted for 583 (8.9%) and 244 (3.7%), respectively. Incidence rates of incisional SSI, organ/space SSI, RTI, UTI, antibiotic‐associated diarrhea, catheter‐related bloodstream infection, drain infection, and bacteremia of unknown origin according to surgical procedure are shown in Figure 2.

Among all PI, 93 (13.2%) were overlapping infections (Table 2). The most common overlapping infections were incisional and organ/space SSI, which were detected in 30 (4.2%) patients.

**Table 2:** Details on overlapping postoperative infections

Overlap pattern	No. of cases
Two overlap	71 (10.1%)
Incisional SSI + organ/space SSI	18
Organ/space SSI + drain infection	6
Organ/space SSI + catheter‐related bloodstream infection	6
Organ/space SSI + RTI	5
Organ/space SSI + bacteremia of unknown origin	5
Organ/space SSI + antibiotic‐associated diarrhea	4
Incisional SSI + antibiotic‐associated diarrhea	3
Organ/space SSI + UTI	3
Catheter‐related bloodstream infection + RTI	3
Incisional SSI + catheter‐related bloodstream infection	2
Incisional SSI + RTI	2
Incisional SSI + UTI	2
RTI + UTI	2
Other	10
Three overlap	16 (2.3%)
Incisional SSI + organ/space SSI + UTI	2
Incisional SSI + organ/space SSI + catheter‐related bloodstream infection	2
Incisional SSI + RTI + UTI	2
Organ/space SSI + RTI + antibiotic‐associated diarrhea	2
Others	8
Four overlap	4 (0.6)
Incisional SSI + organ/space SSI + catheter‐related bloodstream infection + UTI	2
Incisional SSI + organ/space SSI + catheter‐related bloodstream infection + RTI	1
Incisional SSI + catheter‐related bloodstream infection + RTI + bacteremia of unknown origin	1
Five overlap	2 (0.3%)
Incisional SSI + organ/space SSI + RTI + UTI + bacteremia of unknown origin	1
Catheter‐related bloodstream infection + RTI + UTI + antimicrobial‐associated diarrhea + bacteremia of unknown origin	1

RTI, respiratory tract infection; SSI, surgical site infection; UTI, urinary tract infection.

**Figure 3:**
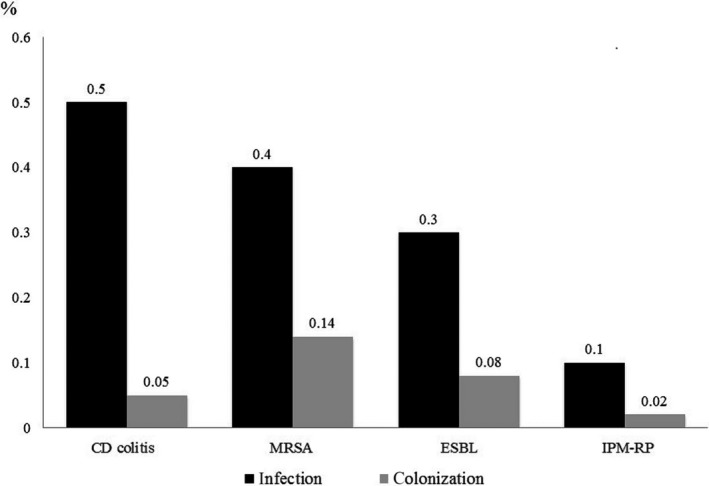
Incidence rates of antimicrobial‐resistant bacterial infection and colonization. CD,* Clostridioides difficile*; ESBL, extended‐spectrum beta‐lactamase‐producing Enterobacteriaceae; IPM‐RP, imipenem‐resistant *Pseudomonas aeruginosa*; MRSA, methicillin‐resistant *Staphylococcus aureus*

Data on AMR bacterial infection and colonization are shown in Figure 3. VRE and MDR‐GN were not isolated during the study period. Infections with AMR bacteria were detected in 81 patients, which included 1.2% of patients after digestive surgery and 11.5% of PI.

In this survey, there was a total of 20 deaths (0.3%), including three (1.1%) in esophageal surgery, six (0.6%) in gastrointestinal surgery, six (0.3%) in colorectal surgery, two (0.4%) in liver surgery, one (0.3%) in pancreatic surgery, and two (1.4%) in surgery for acute peritonitis. Comorbid PI occurred in 19 (95%) of the patients, which included two incisional SSI, 10 organ/space SSI, seven RTI, one UTI, one antibiotic‐associated diarrhea, two catheter‐related bloodstream infections, and two bacteremia of unknown origin. AMR bacteria were isolated from five patients (one CD colitis, two MRSA, and two ESBL‐producing Enterobacteriaceae).

## 4 DISCUSSION

The JPICS‐15 survey, which included data from 6582 patients with SSI, RI, and AMR bacterial infections and colonization, aimed to identify the current status of PI after digestive surgery in Japan. In summary, the PI rate was 10.7%, whereas the rates of SSI and RI were 8.9% and 3.7%, respectively. Of all PI, 13.2% were overlapping infections, with the most common types being incisional and organ/space SSI, which were detected in 4.2% of patients. AMR bacterial infections occurred in 1.2% of patients who underwent digestive surgery and constituted 11.5% of all PI. Conversely, the rate of AMR bacterial colonization was 0.3% in the entire survey cohort.

Surgical site infection is among the most common complications after surgery,^2^ with an incidence rate ranging from 4.0% to 24% depending on the series.^3^, ^4^, ^5^, ^6^, ^7^, ^8^, ^9^, ^10^ According to a national survey conducted in Japan, the incidence of SSI during the period from 2007 to 2016 was 9.6%.^18^ Further analysis showed that the incidence rates of SSI in 2016 were 19.4% for esophageal surgery, 7.9% for gastric surgery, 11.7% for colon surgery, 14.6% for rectal surgery, 14.7% for biliary surgery, 4.9% for appendectomy, and 0.7% for hernia surgery.^18^ Additionally, the National Healthcare Safety Network surveillance showed that the incidence rates of SSI for open and endoscopic surgery were 15.7% and 7.6% for colorectal surgery, 11.2% and 2.2% for liver surgery, 7.2% for biliary surgery (no endoscopic surgery), 17.2% for pancreatic surgery (no endoscopic surgery), and 1.6% for hernia surgery (no endoscopic surgery), respectively.^19^ A European surveillance study reported that the incidence rate of SSI was 9.5% for colorectal surgery and 1.4% for cholecystectomy.^20^

A previous study reported that the incidence of PI was lower with endoscopic surgery than with open surgery.^19^, ^21^ In the present survey, although the background of surgical procedures as well as the classification of benign or malignant disease differed between endoscopic and open surgery, PI were less frequent after endoscopic surgery, especially for esophageal surgery, gastrointestinal surgery, colorectal surgery, and cholecystectomy. Previous studies reported similar results regarding endoscopic surgery versus open surgery in esophageal surgery (23.5% vs 46.7%),^22^ gastrointestinal surgery (1.0% vs 1.8%‐8.9%),^23^, ^24^ colorectal surgery (7.0%‐11.3% vs 15.7%‐25.0%),^25^, ^26^, ^27^ and cholecystectomy (0.6%‐5.9% vs 4.9%‐19.9%).^28^, ^29^, ^30^ In the present survey, there was no incidence of PI after endoscopic esophageal surgery, although the procedures included only esophageal surgery for benign disease, such as esophageal achalasia, hiatus hernia, and selective vagotomy. There was no endoscopic surgery for esophageal cancer in this survey. Also, there was no PI after endoscopic liver surgery, likely because of differences in the surgical procedures between the groups. Endoscopic surgery is a less invasive procedure than open surgery because the wound incision is smaller and exposure of the organs is lower. As a parameter of surgical invasion, blood loss was lower for endoscopic surgery and was associated with a lower incidence of SSI.^31^ Furthermore, endoscopic surgery is associated with less oxidative stress and lower production of inflammatory proteins and cytokines, as compared with open surgery.^32^, ^33^ Therefore, open surgery might exacerbate immune suppression, thereby compromising patients by increasing the risk of PI.^34^ According to these reports, endoscopic surgery is associated with a lower incidence of PI than open surgery. However, a detailed analysis with similar backgrounds of surgical procedures should be carried out to confirm this finding.

Remote infections are important for patient management after surgery. However, no study has yet analyzed the incidence of postoperative RI in detail. In the present survey, the incidence of RI was lower than that reported by studies conducted in other countries,^35^, ^36^, ^37^, ^38^ which can be partially explained by the management of all aspects of postoperative patient care, including the prevention of SSI and RI, directly by the surgeons in Japan. Second, in the present survey, the number of endoscopic surgeries was greater than that of open surgeries as compared with other studies. These situations may have influenced the occurrence of RI.

We also analyzed overlapping PI. The most common postoperative overlapping infections were incisional and organ/space SSI. Therefore, the most important problem regarding the prevention of PI is reducing the incidences of incisional and organ/space SSI. There are several strategies that surgeons can implement to prevent SSI.^39^, ^40^

Antimicrobial‐resistant bacterial infection and colonization, especially after surgery, is a major global clinical problem that is expected to continue to increase in parallel with the increase in the elderly population. The AMR bacterial infection rates in the present survey were lower than those previously reported.^13^, ^14^, ^41^, ^42^, ^43^, ^44^ VRE or MDR‐GN was not isolated from any of the patients who participated in the present survey. However, 11.5% of the PI involved AMR bacteria, indicating that antimicrobial coverage against AMR bacteria should be considered for patients with critical PI. Although a US survey conducted from 1999 to 2003 reported an incidence of CD colitis of 0.52%, recent evidence suggests an increase in this rate.^42^ Conversely, MRSA infections were reported in 0.9% of patients after colorectal surgery,^43^ whereas the frequency of ESBL was significantly increased among the community‐acquired infections.^44^ A UK surveillance study reported that the rate of ESBL was significantly increased from 11.5% in 2007 to 15.4% in 2012 and the rates of ESBL in RTI and UTI were significantly increased from 11.9% to 14.7% and from 9.4% to 16.6%, respectively, among patients receiving intensive care.^44^ Several studies from other countries also found that the rates of VRE were significantly increased.^45^ For example, a UK surveillance study reported that the incidence of SSI as a result of VRE had significantly increased from 0.9% in 2007 to 5.2% in 2016 and the rates of VRE in UTI and bloodstream infections had significantly increased from 2.9% to 9.9% and from 5.9% to 16.7%, respectively, among patients receiving intensive care.^45^, ^46^ Meanwhile, the rate of AMR bacterial colonization was lower than that reported in previous studies, with only 0.3% of the patients affected.^9^, ^15^, ^16^ This is the first report to examine AMR bacterial colonization after digestive surgery; however, the effect of AMR bacterial colonization on outcomes remains unknown. AMR bacterial infection is reportedly associated with a longer duration of hospitalization as well as increased medical costs.^43^, ^46^ Periodic nationwide surveys are important to prevent and reduce the incidence of AMR bacterial infection and colonization. Additionally, the management of surgical procedures and perioperative infections, including antimicrobial therapy, must be periodically evaluated in individual medical centers.

There were several limitations to the present survey. First, this survey was conducted by the Japan Society for Surgical Infection and included 28 voluntary centers, rather than a nationwide survey of PI throughout Japan. Second, this was the first survey to use a specific submission system. However, for ease of data submission, the survey parameters were limited. For example, patient conditions, such as American Society of Anesthesiologists physical status classification and Charlson comorbidity index, were not be evaluated in this survey. Furthermore, for cases without PI, only age, gender, and surgical procedure were registered. Therefore, detailed characteristics, such as patient condition and contamination status, could not be evaluated. Third, the observation period of the present survey was shorter than those of previous surveys and was limited until discharge or 30 days after surgery; thus, readmission cases for PI were not included. Finally, patient distribution was dependent on open surgery or on endoscopic surgery, which differed, especially for esophageal surgery, hepato‐biliary‐pancreatic surgery, and surgery for acute peritonitis. This situation may have been influenced by the incidence of postoperative SSI and RI. However, the main focus of this survey was to identify the actual factors underlying the occurrence of SSI, RI, and AMR infection and colonization after digestive surgery in Japan.

In conclusion, PI, including SSI and RI, in patients after digestive surgery, as well as the incidence of AMR bacterial infection and colonization, were evaluated. Periodic survey of PI, including AMR bacterial surveillance, is necessary for a detailed evaluation of nosocomial infections. Future multicenter prospective randomized control trials based on this surveillance will be useful for a detailed evaluation of PI.

## DISCLOSURE

Conflicts of Interest: Authors declare no conflicts of interest for this article.
